# The image quality and diagnostic accuracy of T1-mapping-based synthetic late gadolinium enhancement imaging: comparison with conventional late gadolinium enhancement imaging in real-life clinical situation

**DOI:** 10.1186/s12968-022-00857-1

**Published:** 2022-04-14

**Authors:** Suji Lee, Panki Kim, Dong Jin Im, Young Joo Suh, Yoo Jin Hong, Byoung Wook Choi, Young Jin Kim

**Affiliations:** Department of Radiology, Research Institute of Radiological Science, Severance Hospital, Yonsei University College of Medicine, 50-1 Yonsei-ro, Seodaemun-gu, 03722 Seoul, Republic of Korea

**Keywords:** Magnetic resonance imaging, Cardiac imaging techniques, Cardiomyopathies

## Abstract

**Backgrounds:**

Synthetic late gadolinium enhancement (LGE) images are less sensitive to inversion time (TI) and robust to motion artifact, because it is generated retrospectively by post-contrast T1-mapping images. To explore the clinical applicability of synthetic LGE, we investigated the image quality and diagnostic accuracy of synthetic LGE images, in comparison to that of conventional LGE for various disease groups.

**Method and materials:**

From July to November 2019, a total of 98 patients who underwent cardiovascular magnetic resonance imaging (CMR), including LGE and T1-mapping sequences, with suspicion of myocardial abnormality were retrospectively included. Synthetic magnitude inversion-recovery (IR) and phase-sensitive IR (PSIR) images were generated through calculations based on the post-contrast T1-mapping sequence. Three cardiothoracic radiologists independently analyzed the image quality of conventional and synthetic LGE images on an ordinal scale with per-segment basis and the image qualities were compared with chi-square test. The agreement of LGE detection was analyzed on per-patient and per-segment basis with Cohen’s kappa test. In addition, the LGE area and percentage were semi-quantitatively analyzed for LGE positive ischemic (n = 14) and hypertrophic cardiomyopathy (n = 13) subgroups by two cardiothoracic radiologists. The difference of quantified LGE area and percentage between conventional and synthetic LGE images were assessed with Mann–Whitney U-test and the inter-reader agreement was assessed with Bland–Altman analysis.

**Results:**

The image quality of synthetic images was significantly better than conventional images in both magnitude IR and PSIR through all three observers (P < 0.001, all). The agreements of per-patient and per-segment LGE detection rates were excellent (kappa = 0.815–0.864). The semi-quantitative analysis showed no significant difference in the LGE area and percentage between conventional and synthetic LGE images. In the inter-reader agreement showed only small systematic differences in both magnitude IR and PSIR and synthetic LGE images showed smaller systematic biases compared to conventional LGE images.

**Conclusion:**

Compared to conventional LGE images, synthetic LGE images have better image quality in real-life clinical situation.

**Supplementary Information:**

The online version contains supplementary material available at 10.1186/s12968-022-00857-1.

## Background

Late gadolinium enhancement (LGE) of myocardium by cardiovascular magnetic resonance imaging (CMR) is a well-established method for detecting myocardial scars or fibrosis in various cardiomyopathies [1–4]. Because conventional LGE sequences utilize the inversion-recovery (IR) technique, successful myocardial nulling is essential for good image quality [5]. Though phase-sensitive IR (PSIR) technique is less sensitive to myocardial nulling time, poor image quality due to inadequate inversion time (TI) are still a concerning factor [6, 7].

T1-mapping-based synthetic LGE imaging, which is derived from the modified Look-Locker IR (MOLLI)-based T1-mapping through retrospective calculation, can be acquired at any theoretical TI on the basis of voxel-by-voxel T1 dataset [5, 8]. Therefore, the optimal nulling time required for better image quality can be estimated [9, 10]. In recent studies, the accuracy of synthetic LGE images has been evaluated in patients with myocardial infarction in comparison to that of conventional LGE images. They showed high sensitivity and specificity of the synthetic IR images for the detection of LGE and excellent agreement with conventional LGE imaging for assessing myocardial scars [11, 12].

However, no study has assessed the comparison of synthetic to conventional LGE images for various disease groups and directly compared image quality between the two image types. Therefore, the purpose of the current study is to investigate the image qualities and diagnostic accuracies of synthetic LGE images compared to that of the conventional LGE technique for various disease groups in real-life clinical situations.

## Methods

### Study population

The Institutional Review Board at the authors’ institution approved this retrospective study and the requirement for informed consent was waived. From July 2019 through November 2019, 100 adult patients who underwent contrast-enhanced CMR, including LGE and post T1-mapping sequences, with suspicion of myocardial abnormality were retrospectively included, regardless of conventional LGE or post T1-mapping sequences’ image qualities. Two patients were excluded because their myocardium was not properly included in the field of view. Thus, a total of 98 patients were included in our final study group.

### CMR protocol and synthetic LGE generation

All patients underwent contrast enhanced CMR with a 3T CMR system (Prisma fit; Siemens Healthineers, Erlangen, Germany) with an 8-channel phased array body coil. LGE and post-contrast T1-mapping sequences are included, which were acquired between 10 and 15 min after injection of 0.2 mml/kg of gadolinium-based contrast agent (Dotarem, Guerbet, Roissy CDG, France). A TI scout sequence was used to determine the TI_0_ for conventional LGE imaging and experienced technologists selected the TI. Conventional LGE sequences were acquired using breath-hold electrocardiogram (ECG) triggered sequences with segmented fast low angle shot (FLASH) readout in standard long and short-axis slices covering the entire left ventricle (LV). Typical parameters were: slice thickness, 8 mm; image acquisition matrix, 192 $\times 135$; in-plane spatial resolution, 1.40 $\times$ 1.40 mm^2^; repetition time, 0.36 and 8.37 ms; echo time, 1.05 ms; bandwidth, 1185 Hz/pixel; and flip angle, 55° and 20°. Post-contrast T1-mapping sequences were acquired using breath-hold ECG triggered MOLLI sequence with a 5(3) 3 protocol at three slices of base, mid, and apex of LV immediately after LGE sequence. For all T1-mapping images, the T1-curve fitting was used, and there was no significant artifact limiting the myocardial evaluation. Typical parameters were: slice thickness, 8 mm; image acquisition matrix, 256 $\times$ 168; in-plane spatial resolution, 1.48 $\times$ 1.48 mm^2^; repetition time, 0.23 ms; echo time, 1.09 ms; bandwidth, 1085 Hx/pixel; and flip angle, 35°.

Synthetic LGE images were generated off-line from acquired post-contrast T1-mapping images using dedicated in-house developed MATLAB-based software (Mathworks, Natick, Massachusetts, USA). After we imported the post-contrast T1-mapping into the software, we generated the synthetic LGE images by using the following equations:

1 $${\text{SI }}\left( {{\text{TI}}} \right)_{{{\text{PSIR}}}} = { 1}{-}{2 } \times {\text{ exp }}\left( { - {\text{2TI}}/{\text{T1}}} \right),$$

and

2 $${\text{SI }}\left( {{\text{TI}}} \right)_{{{\text{MagIR}}}} = \, \left| {{\text{SI }}\left( {{\text{TI}}} \right)_{{{\text{PSIR}}}} } \right|,$$

which were also used in previous studies investigating synthetic LGE. Through the equations, we could generate both magnitude IR and PSIR synthetic images at any theoretical TI [11]. We generated 41 synthetic magnitude-reconstructed and PSIR images between 200 and 600-ms TI at 10-msecincrements (Additional file 1: Fig. S4). The dedicated software also provided a candidate optimal TI image among the synthetic LGE images, which was obtained by multiplying the T1 value of the normal myocardium of the patients by 0.69. With reference to the candidate optimal TI image, we analyzed the synthetic LGE by selecting the most suitable TI image from the image pools by visual assessment.

### Qualitative analysis of image quality and LGE detection rate

Three cardiothoracic radiologists independently analyzed the image quality of conventional and synthetic LGE images for both magnitude IR and PSIR images. In addition, a cardiothoracic radiologist qualitative analyzed the agreement of LGE detection rate between the conventional and synthetic LGE images. All image analyses were performed on three slices (base, mid and apex) of LV at the short axis view, based on the slice obtained from the post-contrast T1-mapping images. The conventional LGE images were analyzed by selecting the same slice at the short axis view. The image quality was analyzed on per-segment basis and the following scoring system was applied: score 1, good image quality available for quantitative evaluation; score 2, fair image quality available for qualitative evaluation; score 3, non-diagnostic image quality. The LGE detection rate, i.e., positive or negative LGE, was analyzed on both per-patient and per-segment basis. Per-segment base analysis was performed by dividing each slice into 4 segments (anterior, lateral, inferior and septal). If it is difficult to determine the presence of LGE due to poor image quality, we evaluated the segment as ‘LGE negative’.

### Semi-quantitative analysis with inter-reader agreement analysis

The semi-quantitative analysis of the LGE area and fraction was performed for ischemic and hypertrophic cardiomyopathy subgroups with LGE positive patients. Because other myocardial disease often show diffuse myocardial fibrosis, which makes it difficult to quantify regional LGE, semi-quantitative analysis was performed for ischemic and hypertrophic subgroups. Two cardiothoracic radiologists independently analyzed the images. They sequentially measured the LGE area on synthetic LGE images after completing measurement on conventional LGE images without referring to the other sequence. Quantification was also performed on 3 slices (base, mid, and apex) of LV at short axis view, in both conventional and synthetic LGE images, using commercially available software (cvi^42^, version 5.11.1, Circle Cardiovascular Imaging Inc., Calgary, Alberta, Canada). After contouring endo- and epi-cardium of the LV manually, the area and percentage of LGE in the contoured myocardium was calculated semi-automatically. The 5-standard deviation (SD) (above the mean remote myocardial signal intensity) technique was used and we manually revised to avoid the boundaries of endo- and epi-cardiac surfaces. The remote non-LGE reference region of each LGE slice was placed adjacent to the region of LGE.

### Statistical analysis

Statistical analyses were performed using MedCalc for Windows (version 19.1.0.0, MedCalc Software, Ostend, Belgium) and R software (version 4.0.2, R Foundation for Statistical Computing, Vienna, Austria). The degree of distribution of image quality score between conventional and synthetic IR images were compared using a chi-square test. The agreement of LGE detection between conventional and synthetic LGE images were analyzed with Cohen’s kappa test. With the subgroups of conventional LGE image quality 1 and 2 groups, the false positive and false negative rates were evaluated. Mann–Whitney U-test was used to compare the quantified area and fraction between conventional and synthetic LGE images. Bland–Altman analysis was used for investigating the quantification and potential systematic differences between the magnitude and synthetic IR techniques, and between the readers. P values less than 0.05 were considered statistically significant.

## Results

A total of 98 patients (57.2 ± 16.7 years, 61% male) underwent contrast-enhanced CMR, including LGE and post-contrast T1-mapping sequences, with suspicion of cardiomyopathy. Diseases of the subjects are listed in Table 1.

**Table 1:** Summary of patient characteristics

Characteristic	Value
Age (y)^a^	57.2 ± 16.7
Sex	
M	60
F	38
Height (cm)^a^	164.5 ± 12.5
Weight (kg)^a^	65.0 ± 12.9
Body mass index (kg/m^2^)	24.4 ± 8.7
Body surface area (m^2^)	1.7 ± 0.2
Disease category	
Ischemic cardiomyopathy	14
Hypertrophic cardiomyopathy	18
Dilated cardiomyopathy	31
Infiltrative cardiomyopathy	4
Inflammatory cardiomyopathy	8
Arrhythmogenic right ventricular cardiomyopathy	1
Unclassified cardiomyopathy	9
Cardiac mass	1
Others^b^	12

Except where indicated, data are numbers of patients (n = 98)

^a^Data are means ± standard deviations

^b^Pericarditis, infective endocarditis, Tetralogy of Fallot, and pulmonary hypertension are included in this category

**Fig. 1:**
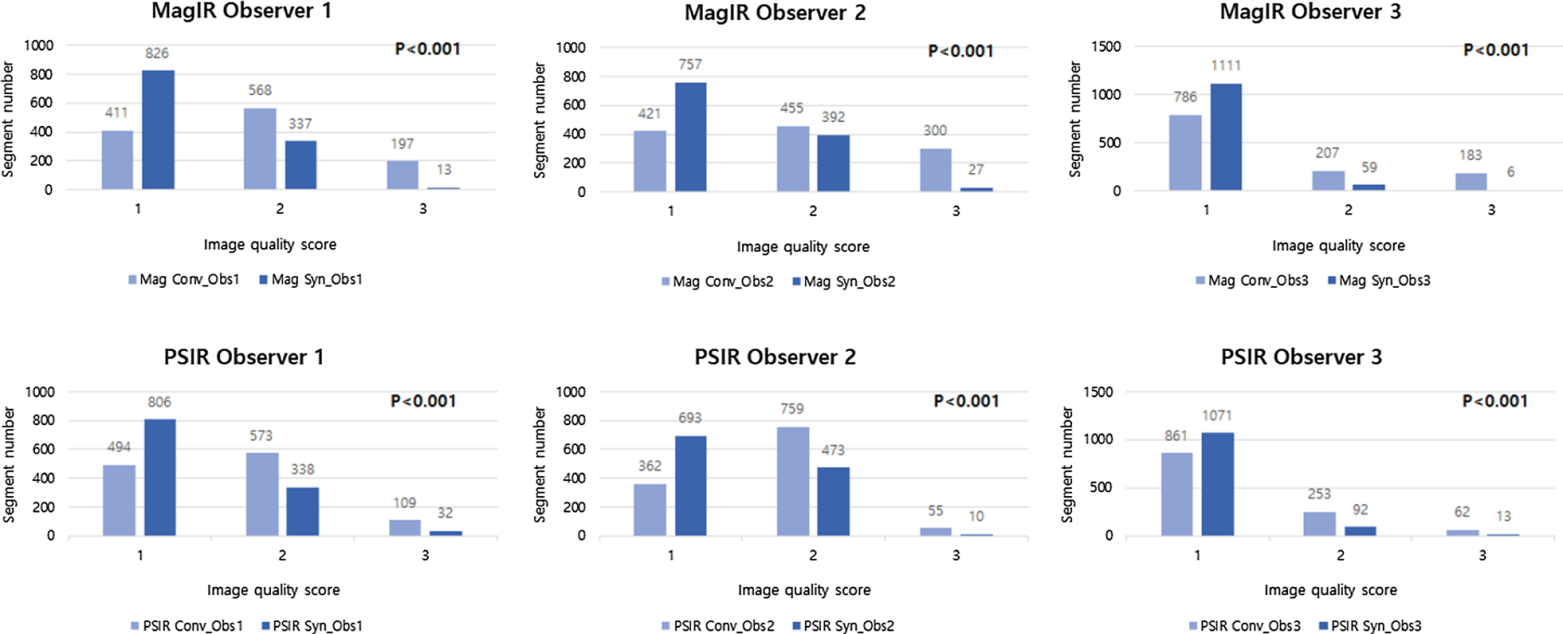
Comparison of image quality score between conventional and synthetic late gadolinium enhancement (LGE) images. Synthetic LGE images showed significantly better image qualities compared to conventional LGE images in both magnitude inversion recovery (IR) and phase sensitive inversion recovery (PSIR) images for all observers (P < 0.001, all). Image quality score 1, good image quality available for quantitative evaluation; score 2, fair image quality available for qualitative evaluation; score 3, non-diagnostic image quality

Upon analysis, the image quality of synthetic LGE images was significantly better than that of conventional LGE images in both magnitude and PSIR imaging for all three observers (P < 0.001, all) (Fig. 1). In the per-patient analysis of agreement between synthetic and conventional LGE images, both magnitude IR and PSIR techniques showed excellent agreement (kappa = 0.815, 90 out of 98 patients, each). In addition, the per-segment analysis also showed excellent agreement in both magnitude IR (kappa = 0.840, 1097 out of 1176 segments) and PSIR (kappa = 0.864, 1109 out of 1176 segments) techniques. (Table 2). The false positive rate and false negative rate of synthetic LGE image obtained with conventional LGE image quality score 1 or 2 subgroup was 6.7% and 1.5% for magnitude IR images, and 3.5% and 1.8% for PSIR images in per-patient analysis, and 11.2% and 3.6% for magnitude IR images, and 7.4% and 5.8% for PSIR images in per-segment analysis.

**Table 2:** Agreement of LGE detection rate between conventional and synthetic IR techniques

		Kappa	95% CI
Per-patient	MagIR	0.815	0.694–0.937
	PSIR	0.815	0.694–0.937
Per-segment	MagIR	0.840	0.807–0.874
	PSIR	0.864	0.832–0.895

*LGE* late gadolinium enhancement, *MagIR* magnitude-reconstructed inversion recovery, *PSIR* phase-sensitive inversion recovery

**Fig. 2:**
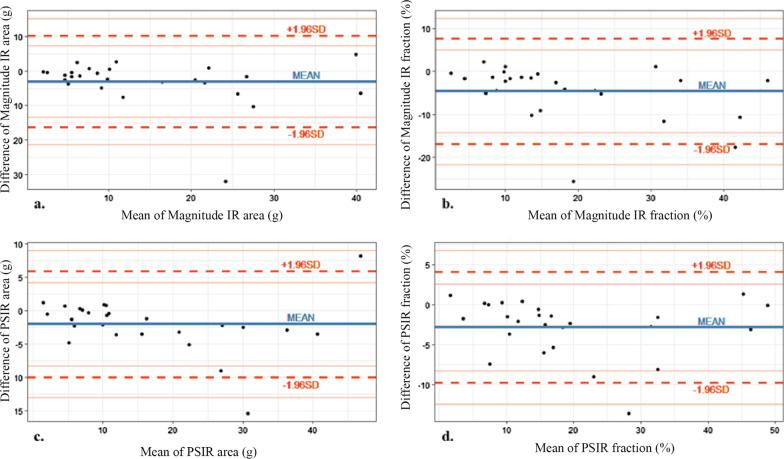
Bland–Altman plots show agreement of (**a**, **c**) LGE area (in square centimeters) and (**b**, **d**) LGE fraction (the percentage of LGE area per total myocardium) with magnitude IR and PSIR techniques. There was no significant difference measured LGE area and percentage in all techniques with small systematic differences

The semi-quantitative analysis showed no significant difference in the LGE area and percentage between conventional and synthetic LGE images, in both magnitude IR and PSIR techniques, with small systematic differences. In addition, though there was no statistical significant difference between measured LGE area and fraction, synthetic LGE showed numerically larger LGE area and fraction than conventional LGE (Table 3; Fig. 2, Additional file 1: Fig. S2).

**Table 3:** Comparison of quantified LGE area and fraction between conventional and synthetic LGE images

	Conventional	Synthetic	P-value*
MagIR			
LGE area (cm^2^)	12.9 ± 10.6	16.0 ± 12.3	0.35
LGE fraction (%)	19.6 ± 13.2	20.6 ± 13.6	0.15
PSIR			
LGE area (cm^2^)	15.3 ± 12.2	17.3 ± 12.8	0.57
LGE fraction (%)	17.9 ± 12.7	20.7 ± 13.2	0.37

Data are mean of the two readers’ measurement, mean ± standard deviations

*LGE* late gadolinium enhancement, *MagIR* magnitude-reconstructed inversion recovery, *PSIR* phase-sensitive inversion recovery

*P-value < 0.05

Analysis of inter-reader agreement of quantified LGE area and percentage of myocardium in LGE-positive ischemic and hypertrophic cardiomyopathy subgroups, there was a small systematic difference and narrow limits of agreement between the readers. Both magnitude IR and PSIR techniques showed smaller systematic biases and more narrow limits of agreement in the synthetic LGE images, compared to that of the conventional LGE images (Table 4, Additional file 1: Fig. S1).

**Table 4:** Inter-reader agreement of quantified LGE area and fraction

	Inter-reader agreement^a^	
Technique	LGE area (cm^2^)	LGE fraction (%)
Synthetic MagIR	− 0.1 (− 7.2, 7.0)	− 0.1 (− 6.9, 6.8)
Synthetic PSIR	− 0.3 (− 5.8, 5.3)	0.3 (− 3.9, 4.5)
Conventional MagIR	1.0 (− 3.4, 5.3)	1.3 (− 4.3, 6.9)
Conventional PSIR	− 0.3 (− 6.9, 6.2)	0.4 (− 5.5, 6.3)

*LGE* late gadolinium enhancement, *MagIR* magnitude-reconstructed IR, *PSIR* phase-sensitive IR

^a^Data are biases and were obtained with Bland–Altman analysis. Numbers in parentheses are the 95% limits of agreement

## Discussion

Our study demonstrates that T1-mapping based synthetic LGE images are better in quality than conventional LGE images. There was excellent agreement between synthetic and conventional LGE images for both qualitative and semi-quantitative analysis. Moreover, the analysis of inter-reader agreement showed only a small systematic difference between the readers, and smaller biases and more narrow limits of agreement were shown in synthetic LGE images compared to conventional LGE images.

Since T1-mapping based synthetic LGE imaging was proposed as a novel technique to evaluate the myocardium, several studies have demonstrated the compatibility of its diagnostic accuracy with that of conventional LGE imaging. However, there have been no studies systematically comparing the image quality between synthetic and conventional LGE images. Furthermore, the subjects in previous studies analyzing the diagnostic accuracy of synthetic LGE images were limited to patients with ischemic cardiomyopathy or animals [9–12, 14]. Therefore, the novelty of this study is that it is the first study investigating and verifying better image quality of synthetic LGE images compared to conventional LGE images, with all patient groups suspected of myocardial disease.

**Fig. 3:**
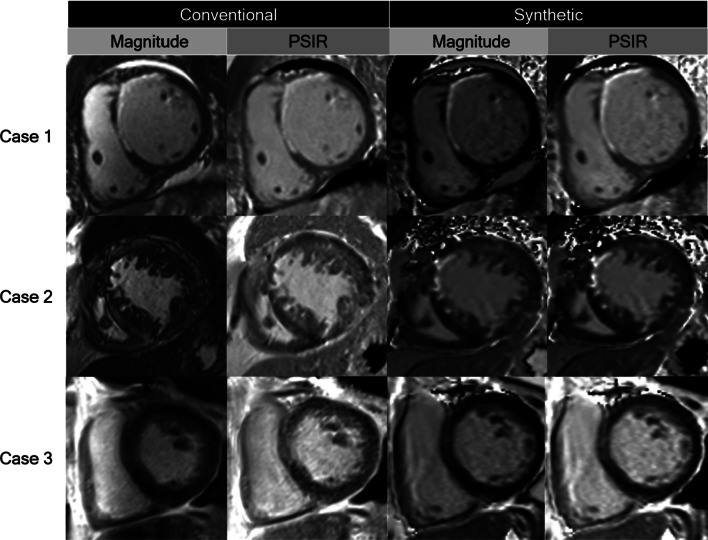
Representative cases. Case 1, A case of 42 year-old man with an ischemic cardiomyopathy and anteroseptal subendocardial late gadolinium enhancement (LGE). All sequences showed good image qualities. Case 2, A case of 80 year-old man with an ischemic cardiomyopathy. Subendocardial LGE at anteroseptal left ventricular (LV) wall is poorly delineated in conventional magnitude inversion recovery (IR) image due to inadequate nulling time. Case 3, A case of 70 year-old man with a dilated cardiomyopathy. Mid-wall LGE at septal LV wall is poorly delineated in conventional LGE images due to arrhythmia

T1-mapping based synthetic LGE images have an advantage in artifact reduction, by retrospectively selecting adequate myocardial nulling time [11]. The optimal TI can be selected to best visualize the contrast of normal myocardium, late gadolinium-enhanced myocardium, and blood pool [10]. This could prevent spoiling the image due to inadequate myocardial nulling time, so synthetic LGE images are less dependent on the technician’s experience. In our study, among the 98 cases of conventional images, 18 cases of magnitude IR image and one case of PSIR image show non-diagnostic image quality due to inadequate nulling time. Also, when we compared the TI of conventional and synthetic LGE images, we found that the TI of synthetic LGE (Magnitude IR, 464 ± 69; PSIR 369 ± 86) was generally higher than that of conventional LGE (305 ± 39 for both Magnitude IR and PSIR), and the range of TI was wider in synthetic LGE (Magnitude IR, 290-580 ms; PSIR 230–550 ms) than conventional LGE (225–410 ms for both Magnitude IR and PSIR). The wider range of TI in synthetic LGE could imply that various optimal TIs for each patient are not well reflected in conventional LGE images. Therefore, compared to the optimal TI of synthetic LGE, which was retrospectively selected, the TI of conventional LGE might be underestimated by technicians (Additional file 1: Fig. S3). In addition, synthetic LGE images have little ghost artifacts compared to conventional LGE images. Conventional LGE imaging requires repeated IR pulses with a relatively short recovery time. For tissues that do not take up contrast or have long T1 times, such as pericardial effusion, the time between IR pulses is insufficient to recover longitudinal magnetization; therefore, the oscillations occur between imaging segments [15]. In contrast, the MOLLI sequence, which is the original image of synthetic LGE, uses only two to three IR pulses and ensures sufficient T1 recovery time, resulting in less artifacts for tissues with long T1 time [16]. Furthermore, synthetic LGE images show less motion artifacts (patient’s motion, arrhythmia, etc.) than conventional LGE images, as they are based on single-shot imaging, which is less affected by motion. Also, the artifacts are further reduced by a curve-fitted imaging technique [16, 17]. When we reviewed electronic medical records of five cases in which the image quality score differs the most between conventional and synthetic LGE images, four of them were due to motion artifact (e.g., breathing difficulty, poor cooperation by hearing loss, patient movement, and arrhythmia) and the other case was due to inadequate myocardial nulling time (Fig. 3). In addition, there was 9 and 13 cases that showed worse image quality in synthetic LGE image than conventional LGE image for magnitude IR and PSIR, each. These cases had no LGE or showed only regional LGE without diffuse myocardial infiltration.

In the results of the semi-quantitative analysis, the inter-reader bias of synthetic magnitude IR and PSIR images were smaller than that of conventional magnitude IR and PSIR images. The inter-reader bias was the largest in conventional magnitude IR image in both measured area and fraction of myocardium among the all techniques. Though there was no significant difference of quantified LGE area and fraction between conventional and synthetic LGE images, the inter-reader agreements were higher for synthetic LGE images. The result of inter-reader agreement could be explained in the same way with the results of image quality. In the analysis of image quality, the synthetic PSIR image showed the most score 1 (good image quality) segments, while the conventional magnitude IR image showed the most score 3 (non-diagnostic image quality) segments. As already known, the PSIR technique reduces the need for precise choice of inversion time and provides more consistent image quality than the magnitude IR technique [6]. Better image qualities of conventional PSIR images than that of conventional magnitude IR images, contrary to the overall good image qualities of synthetic magnitude IR and PSIR images, could explain the higher agreement of conventional and synthetic PSIR images than that of conventional and synthetic magnitude IR images. In addition, in the results of the semi-quantitative analysis, synthetic LGE showed numerically larger measured LGE area and fraction than conventional LGE, though there was no statistical significance. The lesions that are not detected in conventional LGE image due to inadequate nulling time and patchy LGE that was detected more sensitively in synthetic LGE due to the advantage of retrospectively acquired images at various inversion times might be the reason why synthetic LGE showed numerically larger LGE area and fraction.

Even though the excellence of T1-mapping sequence for the evaluation of diffuse myocardial disease has been reported, the diagnostic value of LGE images and advantages of intuitive visual assessment for regional fibrosis cannot be ignored. In the recent trends where the quantitative myocardial characterization is available through native T1 value and extracellular volume fraction (ECV), both visual (LGE) and quantitative (native T1 value and ECV fraction) myocardial assessment is available with shorter scan time and better image quality, if the conventional LGE sequence is replaced with synthetic LGE image using post-T1-mapping sequence.

### Limitations

Our study has several limitations. First, our sample size was relatively small, from a single institution, and scanned with a single CMR scanner. Further studies with larger datasets including wider variety of pathologies from multicenter data could demonstrate the better image quality of synthetic LGE more clearly. Second, the investigators were not blinded to the image type in qualitative and semi-quantitative analysis. However, the image characteristics are different between the conventional and synthetic LGE images, it is difficult to blind the image type to the cardiothoracic experts. Third, because we acquired the post T1-mapping sequences after LGE sequences, there could be a delay time difference between the evaluated images. It could affect the difference of TI between conventional and synthetic LGE, which is generally higher in synthetic LGE images. Finally, we did not evaluate the inter-reader agreement of detection rate between the techniques. However, the subgroup semi-quantitative analysis showed no significant difference between the measured area and percentage of myocardium as well as smaller systematic bias of inter-reader agreement at synthetic LGE images. Although the semi-quantitative analysis of myocardial LGE was also somewhat subjective for several reasons, such as partial volume effect affecting border zone, the relatively consistent result of reduced bias in synthetic technique could defend it [18].

## Conclusions

Synthetic LGE images have better image quality and are comparable with conventional LGE images for the detection and quantification of LGE. With further validation using larger datasets and a wider range of pathologies, synthetic LGE images may replace the conventional LGE images if T1-mapping sequence covers the entire LV.

## Supplementary Information

**Additional file 1**: **Fig. S1. **Bland-Altman plots for the inter-reader agreement of quantified area and percentage of myocardium in LGE-positive ischemic and hypertrophic cardiomyopathy subgroups. There was a small systematic difference between the two readers. **Fig. S2**. Distribution of quantified LGE fraction and area between conventional and synthetic magnitude IR and PSIR images. **Fig.S3. **Bland-Altman plots for the inversion time (TI) between conventional and synthetic LGE images in (a) Magnitude IR and (b) PSIR images. **Fig. S4. **Reconstructed synthetic LGE PSIR images between inversion times of 200 and 600 msec with 10 msec increments. The selected image for myocardial evaluation was TI of 340ms (yellow box).

## Data Availability

Applicable. Data: available for joint research by e-mailing. In-house developed software: available for research by e-mailing

## Abbreviations

CMR: Cardiovascular magnetic resonance
ECG: Electrocardiogram
ECV: Extracellular volume fraction
IR: Inversion recovery
LGE: Late gadolinium enhancement
LV: Left ventricle/left ventricular
MOLLI: Modified Look Locker inversion recovery
PSIR: Phase sensitive inversion recovery
TI: Inversion time
