# Supplementary material for: The image quality and diagnostic accuracy of T1-mapping-based synthetic late gadolinium enhancement imaging: comparison with conventional late gadolinium enhancement imaging in real-life clinical situation
Source: J Cardiovasc Magn Reson. 2022 Apr 14;24:28. doi: 10.1186/s12968-022-00857-1 (PMC9008937; doi:10.1186/s12968-022-00857-1)
Supplement: Supplementary file 1 — Additional file 1: Fig. S1. Bland-Altman plots for the inter-reader agreement of quantified area and percentage of myocardium in LGE-positive ischemic and hypertrophic cardiomyopathy subgroups. There was a small systematic difference between the two readers. Fig. S2. Distribution of quantified LGE fraction and area between conventional and synthetic magnitude IR and PSIR images. Fig.S3. Bland-Altman plots for the inversion time (TI) between conventional and synthetic LGE images in (a) Magnitude IR and (b) PSIR images. Fig. S4. Reconstructed synthetic LGE PSIR images between inversion times of 200 and 600 msec with 10 msec increments. The selected image for myocardial evaluation was TI of 340ms (yellow box). [file 12968_2022_857_MOESM1_ESM.pdf]

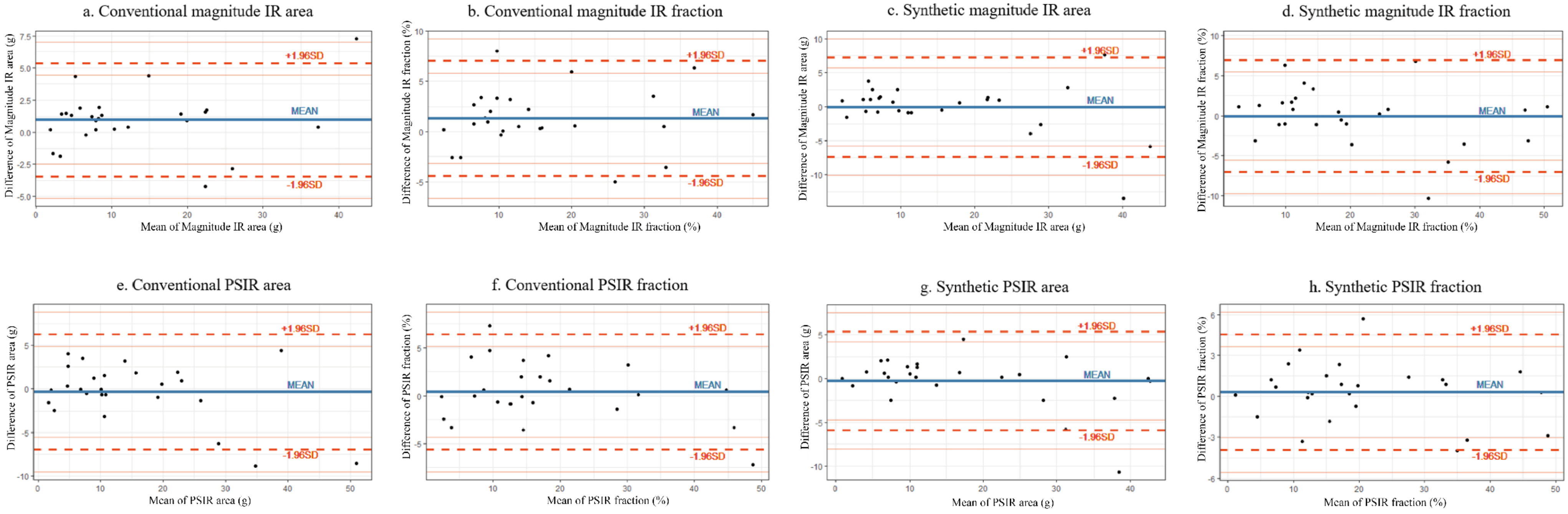

Figure S1: Bland-Altman plots for the inter-reader agreement of quantified area and percentage of myocardium in LGE-positive ischemic and hypertrophic cardiomyopathy subgroups. There was a small systematic difference between the two readers.

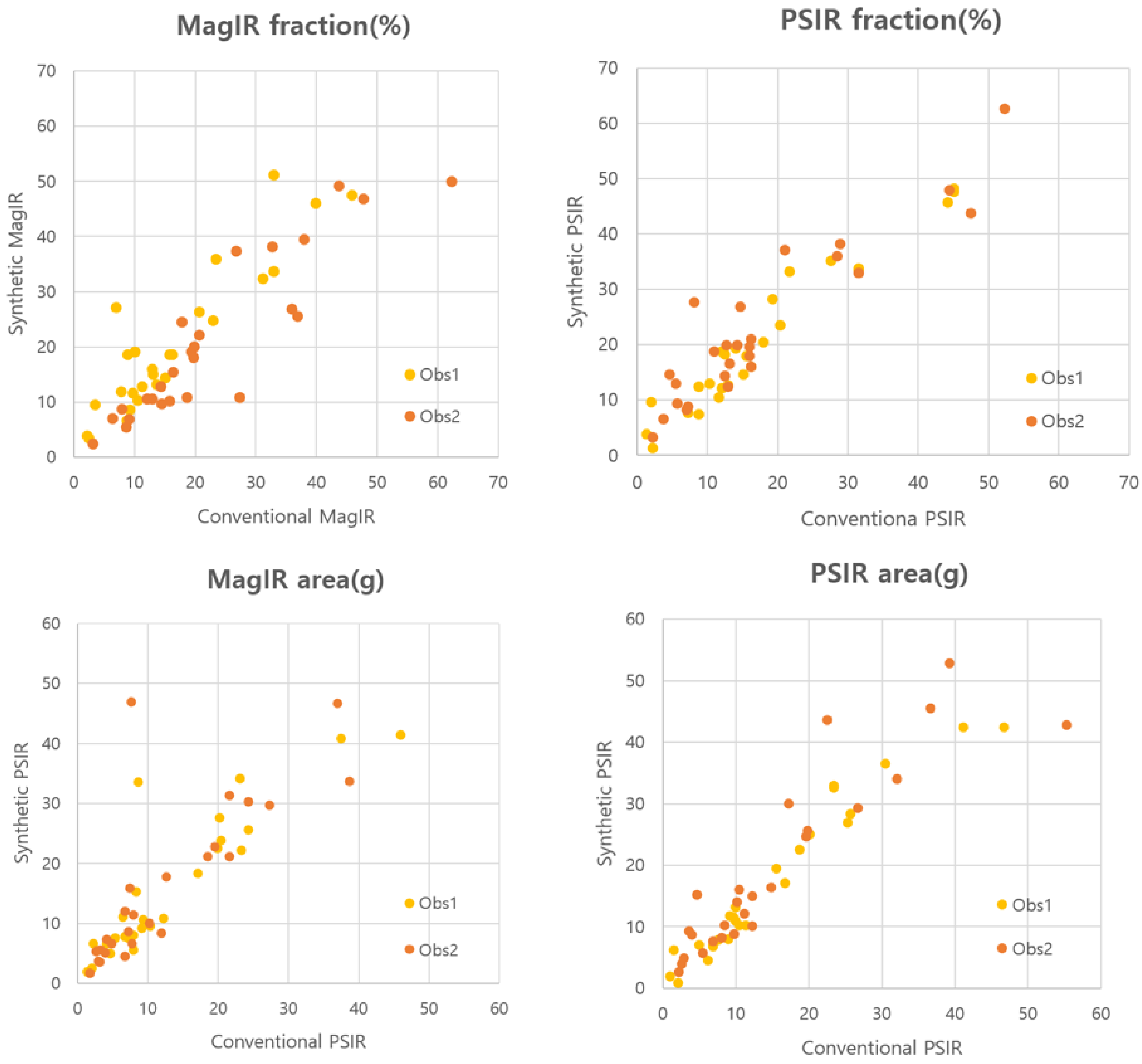

Figure S2. Distribution of quantified LGE fraction and area between conventional and synthetic magnitude IR and PSIR images.

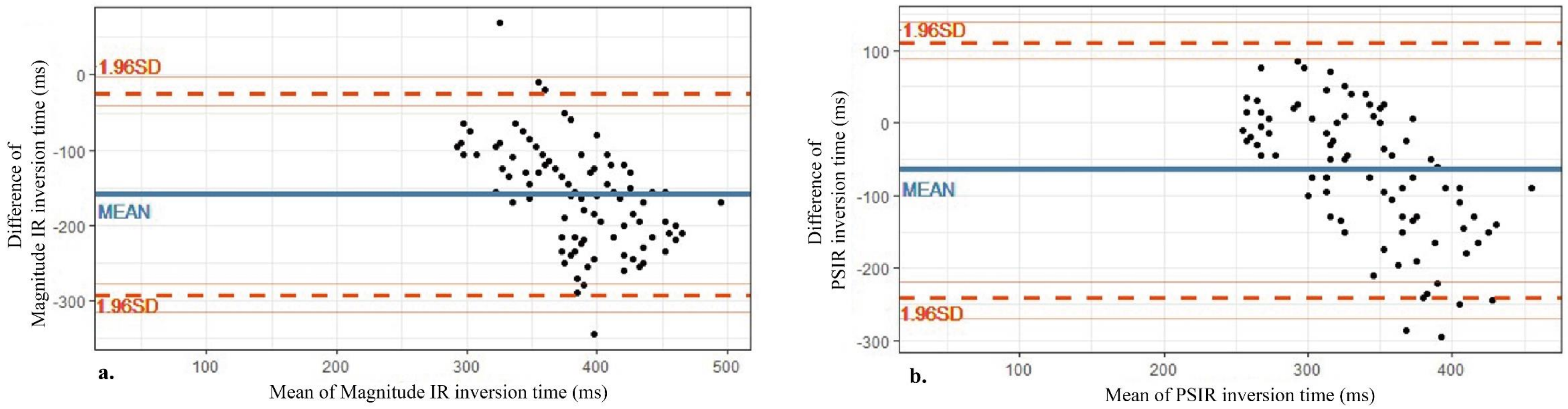

Figure S3. Bland-Altman plots for the inversion time (TI) between conventional and synthetic LGE images in (a) Magnitude IR and (b) PSIR images.

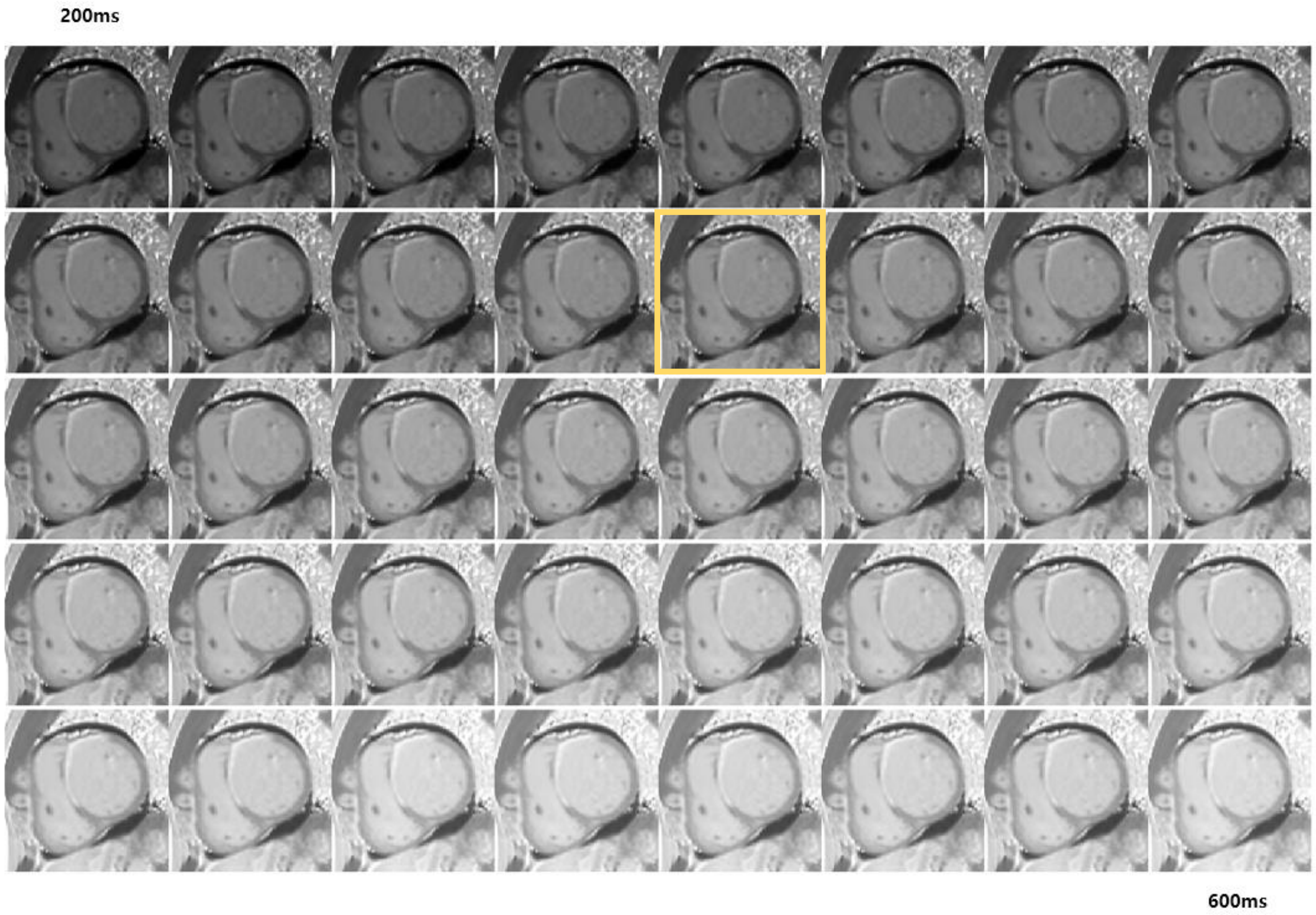

Figure S4. Reconstructed synthetic LGE PSIR images between inversion times of 200 and 600 msec with 10 msec increments. The selected image for myocardial evaluation was TI of 340ms (yellow box).
